# Long-term consumption of an obesogenic high fat diet prior to ischemia-reperfusion mediates cardioprotection via Epac1-dependent signaling

**DOI:** 10.1186/s12986-016-0147-1

**Published:** 2016-11-28

**Authors:** F. Edland, A. Wergeland, R. Kopperud, K. S. Åsrud, E. A. Hoivik, S. L. Witsø, R. Æsøy, L. Madsen, K. Kristiansen, M. Bakke, S. O. Døskeland, A. K. Jonassen

**Affiliations:** 1 Department of Biomedicine, Faculty of Medicine and Dentistry, University of Bergen, Bergen, Norway; 2 Department of Biology, University of Copenhagen, Copenhagen, Denmark; 3 National Institute of Nutrition and Seafood Research, Bergen, Norway; 4 Faculty of Health Science and Medicine, Norwegian University of Science and Technology, NTNU, Trondheim, Norway

**Keywords:** Myocardial ischemia, Reperfusion injury, PKA, Epac, cAMP, Infarct size, High fat diet, Cardiac function, Obesity, Heart

## Abstract

**Background:**

Obesity is still considered a risk factor for cardiovascular disease, although more recent knowledge also suggests obesity to be associated with reduced morbidity and mortality - the “obesity paradox”. This study explores if long-term feeding of an obesogenic high fat diet renders the myocardium less susceptible to ischemic-reperfusion induced injury via Epac-dependent signaling.

**Methods:**

Wild type (wt), Epac1 (Epac1^−/−^) and Epac2 (Epac2^−/−^) deficient mice were fed a high fat (HFD) or normal chow diet (ND) for 33 ± 1 weeks. Six experimental groups were included: (1) control wt ND (wt *ND*), (2) control wt HFD (wt *HFD*), (3) Epac1^−/−^ mice on ND (Epac1^−/−^ *ND*), (4) Epac1^−/−^ mice on HFD (Epac1^−/−^ *HFD*), (5) Epac2^−/−^ mice on ND (Epac2^−/−^ *ND*), and (6) Epac2^−/−^ mice on HFD (Epac2^−/−^ *HFD*). Isolated ex vivo mice hearts were perfused in a constant pressure Langendorff mode, and exposed to 30min of global ischemia (GI) and 60min of reperfusion. Endpoints were infarct size and functional recovery.

**Results:**

All groups fed a HFD presented with significantly enhanced body weight, visceral fat content and reduced glucose clearance compared to corresponding ND groups. Although the HFD cohorts presented with an overall comparable systemic capability to clear glucose, the Epac1^−/−^ HFD group presented with glucose levels slightly above the human diabetes criteria at the end of the intraperitoneal glucose tolerance test (ipGTT). Moreover, the HFD significantly reduced infarct size in both wild type (wt *HFD* 41.3 ± 5.5% vs. wt *ND* 58.0 ± 9.8%, *p* < 0.05) and Epac2^−/−^ cohorts (Epac2^−/−^ *HFD* 34.4 ± 7.2% vs. Epac2^−/−^ *ND* 56.5 ± 3.8%, *p* < 0.05). Interestingly, however, the HFD did not reduce infarct size in Epac1^−/−^ deficient mice hearts (Epac1^−/−^ *HFD* 65.1 ± 5.1% vs. Epac1^−/−^ *ND* 56.1 ± 3.5%, ns.).

**Conclusion:**

Epac1-dependent signaling is involved in mediating the cardioprotection afforded by long-term feeding of an obesogenic high fat diet in mice hearts.

## Background

Obesity serves as a prevalent risk factor for cardiovascular disease, and chronic consumption of fat has traditionally been associated with increased risk for arteriosclerotic cardiovascular disease such as acute myocardial infarction (AMI) [1–5]. More recent observations have, however, suggested that human obesity combined with ischemic heart disease is associated with reduced morbidity and mortality, the so-called “obesity paradox” [6–10]. Despite intense investigations, the precise mechanism(s) underlying this paradox is complex and remains to be fully elucidated.

Recent data imply that an obesogenic high fat diet enhance myocardial tolerance against ischemic-reperfusion induced injury (IR), but this cardioprotection was not associated with activation of ERK p44/p42 or PKB/Akt dependent RISK signaling (reperfusion injury salvage kinase pathway) [11], a pathway widely accepted to mediate protection against reperfusion injury [12, 13]. Furthermore, a recent study by Salie et al. suggests that hearts protected by obesogenic diets are unresponsive to additional cardioprotective conditioning maneuvers, such as ischemic preconditioning (IPC) or β-adrenergic preconditioning (β-PC), indicating that the hearts were maximally protected by the diets [11]. Both IPC and β-PC may elicit protection against IR induced injury via the β-adrenergic cAMP - PKA signal transduction pathway [14–16]. Whether β-adrenergic signaling is involved in mediating high fat diet induced cardioprotection is currently unknown.

Most of the cardiac effects of β-adrenergic cAMP signaling were assigned to PKA, until de Rooij et al. [17] and Kawasaki et al. [18] identified the Epac proteins (Exchange Proteins directly Activated by cAMP; Epac1 and Epac2; also called cAMPGEFs), which have proven to be important downstream mediators of cAMP in multiple physiological pathways. When Epac binds cAMP, it acts as an exchange factor for the small G-proteins Rap1 and Rap2, catalysing the exchange of bound GDP for GTP, subsequently activating a wide array of effectors [19]. In the heart, Epac has been shown to play a role in Ca^2+^-handling and excitation-contraction coupling via phospholipase C, CaMKII, and PKC dependent signaling [20–23]. Furthermore, studies aiming at direct pharmacologic activation of Epac using the cAMP analogue 8-CPT (8-pCPT-2’-O-Me-cAMP), that discriminates between PKA and Epac in vitro [24], have incriminated Epac in cardiac hypertrophy and heart failure [25, 26], in addition to protecting from IR-induced kidney injury [27]. Whether Epac-dependent signaling may contribute towards elevated myocardial tolerance against IR- induced injury in the heart is currently unknown and needs further elucidation.

In the present study, we assessed whether long-term feeding of an obesogenic high fat diet may enhance tolerance against ischemic-reperfusion induced injury in the murine heart. Furthermore, we also aimed to delineate whether Epac, a downstream mediator of β-adrenergic/cAMP mediated signaling, is involved in diet-induced cardioprotection. Due to the low stability of cAMP analogs (including 8-pCPT-2’-O-Me-cAMP) and Epac inhibitors in vivo (recently reviewed in [28]), we employed mice deficient in Epac1 and Epac2 in this study. Here we report that wild type mice exposed to long-term consumption of a HFD exhibited enhanced cardiac tolerance against IR-induced injury without any adverse effects on cardiac function. Importantly, HFD induced protection was lost in Epac1 deficient mice, implicating an important role for Epac1- dependent signaling in HFD induced cardioprotection.

## Methods

### Animals

Fifty-two female mice of the following genotypes were included in this study: 24 wild type (wt), 12 Epac1^−/−^ and 16 Epac2^−/−^. Artificial lighting was maintained on a 12:12-h light-dark cycle, room temperature kept at 22 ± 1 °C and the animals provided with water and food *ad libitum*. The Epac-deficient mouse models used in this study are described in detail elsewhere [29]. In short, *loxP* sites were inserted by homologous recombination into the genes encoding Epac1 (*RapGEF3*) and Epac2 (*RapGEF4*) flanking exons 7–10 in *RapGEF3* and exons 12–13 in *RapGEF4*. These exons encode the cAMP-binding domain in both proteins. A Neomycin cassette flanked by *frt*-sites were used for screening purposes and thereafter excised. Epac1^floxed/floxed^ and Epac2^floxed/floxed^ mice were generated at the Mouse Clinical Institute, Strasbourg, France. In this study, mice globally deleted for Epac1 (Epac1^−/−^) or all isoforms of Epac2 (Epac2^−/−^) were produced by crossing floxed animals with mice expressing Cre recombinase from the cytomegalovirus (CMV)-promoter. The mice were bred against a C57BL/6JBomTac (Taconic, Denmark) genetic background for at least ten generations by the start of the study. Epac1^−/−^ and Epac2^−/−^ mice are healthy and apparently indistinguishable from wt mice under standard housing. The wild type C57BL/6JBomTac animals included were littermates and commercial mice (Taconic, Denmark). Both Epac1 and Epac2 are expressed in hearts of wild type (wt) mice, and there are no compensatory increase in expression of cardiac Epac1 in Epac2^−/−^ mice or Epac2 in Epac1^−/−^ mice in our mice models (results not shown).

### Biochemical analyses/Biometric analysis

Intraperitoneal glucose tolerance tests (ipGTT) were performed at baseline (before the start of the feeding experiments) and at 28 weeks into the feeding experiment. In brief, animals were fasted overnight (15 h) before administration of an intraperitoneal glucose solution (D-glucose, 2 g/kg) followed by blood sampling from the exposed saphenous vein. Blood glucose concentration was measured at 0, 15, 30, 60, 90, 120 and 240 (where applicable) minutes with a glucometer (Precision Xceed, Abbott Laboratories). At the end of the feeding experiment, the peri-gonadal (gWAT) and peri-renal (rWAT) fat was removed by dissection, performed similarly in all mice, to obtain a proportional (relative) estimate of the amount of visceral retroperitoneal fat in the animals. Similarly, the inguinal fat was removed to represent the subcutaneous fat depot.

### Feeding protocol and experimental groups

**Fig. 1:**
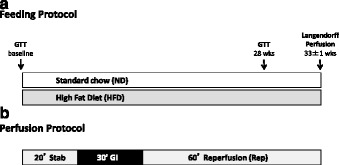
Feeding regiment and experimental protocol. **a** Wild type (wt), Epac1^−/−^ and Epac2^−/−^ mice were divided into 6 groups exposed to either a normal chow diet (ND) or a high fat diet (HFD) for 33 ± 1 weeks. One intraperitoneal glucose tolerance test (ipGTT) was performed before start of the feeding experiment (baseline) and another 28 weeks into the feeding protocol. **b** At the end of the feeding experiment, the hearts were excised and mounted in a Langendorff perfusion system. Hearts were stabilized for 20min, before being subjected to 30min global ischemia (GI) and 60min of reperfusion (Rep)

All mice received a regular chow diet (ND), until they were randomly divided into one of two feeding regimes for 33 ± 1 weeks: regular chow diet (ND) (Special Diets Services, RM1 801151; 75.1% calories from carbohydrate, 17.5% calories from protein and 7.4% calories from fat) vs. high fat diet (HFD) (Research Diets, D12492; 20% calories from carbohydrate, 20% of calories from protein and 60% calories from fat). Four animals were housed in each cage, giving rise to a total of six groups (Fig. 1a): (1) wild types (wt) subjected to a ND throughout the experiment (wt *ND*), (2) wild types (wt) subjected to a HFD (wt *HFD*), (3) Epac1^−/−^ animals subjected to a ND (Epac1^−/−^ *ND*), (4) Epac1^−/−^ subjected to a HFD (Epac1^−/−^ *HFD*), (5) Epac2^−/−^ animals subjected to a ND (Epac2^−/−^ *ND*), and (6) Epac2^−/−^ subjected to a HFD (Epac2^−/−^ *HFD*).

### Isolated Langendorff mouse heart perfusion

Mice were intraperitoneally heparinized (500 IU) and anesthetized with pentobarbital (50 mg/kg). The hearts were excised from the thoracic cavity, placed in ice-cold Krebs-Henseleit buffer (KHB) containing (mM): 118 NaCl, 4.7 KCl, 1.8 CaCl_2_ x 2 H_2_O, 1.2 KH_2_PO_4_, 1.2 MgSO_4_ x 7 H_2_O, 25.2 NaHCO_3_, 11.0 d-Glucose. Aorta was immediately cannulated and the mice hearts retrogradely perfused in Langendorff modus with warm (37 °C), gassed (95% O_2_/5% CO_2_, pH 7.4) KHB at constant pressure (70 mmHg). To monitor heart rate (HR) and ventricular pressures, a fluid-filled balloon-tipped pressure catheter connected to a high performance data acquisition system (PowerLab 8/30, Chart Pro software-MLS250) via a hydrostatic pressure transducer (SP844, Memscap, Norway), was inserted into the left ventricle via the left atrium. The left ventricular end-diastolic pressure (LVEDP) was set to 5–10 mmHg at the start of the experiment. Left ventricular developed pressure (LVDP = LVSP − LVEDP), maximum/minimum derivative of left ventricular pressure development (LVdP/dt_max_ and LVdP/dt_min_; mmHg/s), rate pressure product (RPP = HR x LVDP (bpm x mmHg); a measure of internal workload or hemodynamic response, an indicator of myocardial oxygen demand (MVO2)) and coronary flow (CF; ml/min) was recorded throughout the experiment. The Langendorff perfused ex vivo mice hearts were subjected to an experimental protocol of 110min; 20min of stabilization followed by 30min of global ischemia (GI) and 60min of reperfusion (Fig. 1b). Hearts with aortic cannulation time > 3min, CF < 1 ml/min and > 5.0 ml/min, LVSP < 70 mmHg or > 20min of irreversible arrhythmias (asystole or ventricular fibrillation) during reperfusion were excluded from the study. A total of 9 hearts were excluded for the following reasons: 3 hearts due to CF > 5.0 ml, 2 due to LVSP < 70 mmHg, 3 due to arrhythmias, and one due to improper staining.

### Infarct size determination

At the end of the perfusion protocol the mice hearts were weighed, frozen at −4 °C for 1 h, and cut in 1 mm thick slices using a stainless steel matrix slicer (Alto, Angthos). The heart slices were thereafter stained for 12min at 37 °C using 1% triphenyltetrazolium chloride (TTC; Sigma Chemicals) in phosphate buffer (pH 7.4) to demarcate the viable from non-viable tissue. To enhance the contrast of the stain, the slices were submerged in 4% formalin for 1min, and subsequently scanned at high resolution (2400 dpi, Epson Perfection 4490 Photo). Photoshop-based image analysis (Adobe Photoshop) was used in combination with a digitalised tracing board (Cintiq 13”HD, Wacom) to determine the infarct size, which was expressed as percent of the area at risk (risk volume), which for global ischemia is the whole heart (total ventricular volume, minus cavity spaces).

### Statistics

All statistical analysis was performed using IBM SPSS Statistics, and the results expressed as mean ± standard error of the mean (SEM). Group differences regarding weights, AUC, infarct size and hemodynamic recordings were tested by one-way analysis of variance (ANOVA) combined with Fisher’s post-hoc test. p values ≤ 0.05 was considered statistically significant.

## Results

### Biometric parameters

The three mice cohorts subjected to long-term high fat feeding (HFD) presented with significantly (*p* < 0.05) higher body weight (BW), HW/BW ratio, total and visceral fat content than the age-matched controls fed a normal chow diet (ND) (Table 1). Furthermore, the heart weight of the Epac1^−/−^ *HFD* group was significantly higher than the corresponding Epac1^−/−^ *ND,* the wt *HFD* and the Epac2^−/−^ *HFD* (Table 1). Despite this, the left ventricular (LV) volume did not differ between the groups. Moreover, the HW/BW of the Epac1^−/−^ *ND* were significantly higher than wt *ND* and the Epac2^−/−^ *ND*.

**Table 1:** Biometric data

	Wt *ND*	Wt *HFD*	Epac1^−/−^ *ND*	Epac1^−/−^ *HFD*	Epac2^−/−^ *ND*	Epac2^−/−^ *HFD*
N	8	16	6	6	6	10
BW (g)	27.5 ± 1.6	44.5 ± 2.0^&^	24.2 ± 0.5	48.4 ± 1.1^&^	24.7 ± 0.6	38.8 ± 3.0^&^
HW (mg)	146.3 ± 8.2^¤^	162.2 ± 6.7^¤^	154.3 ± 8.6^¤^	197.7 ± 6.0^&^	126.5 ± 7.2^¤^	137.4 ± 5.3^¤^
HW/BW (mg/g)	5.3 ± 0.2^€^	3.7 ± 0.2^&^	6.4 ± 0.4	4.1 ± 0.2^&^	5.2 ± 0.3^€^	3.6 ± 0.2 &
LV volume (mm^3^)	108.6 ± 6.7	117.5 ± 5.2	96.8 ± 4.8	124.7 ± 8.8	96.0 ± 5.4	104.9 ± 2.7
Total fat (g)	1.7 ± 0.3	7.7 ± 0.5^&^	1.2 ± 0.1	8.6 ± 0.4^&^	1.1 ± 0.1	6.9 ± 0.7^&¤^
iWAT (g)	0.4 ± 0.1	2.0 ± 0.1^&^	0.3 ± 0.1	1.8 ± 0.1^&^	0.4 ± 0.1	1.9 ± 0.2^&^
rWAT (g)	0.5 ± 0.1	2.1 ± 0.2^&^	0.3 ± 0.1	2.5 ± 0.2^&^	0.3 ± 0.1	2.0 ± 0.2^&^
gWAT (g)	0.8 ± 0.2	3.6 ± 0.3^&^	0.6 ± 0.1	4.3 ± 0.2^&^	0.5 ± 0.1	3.0 ± 0.3^&¤^
rWAT + gWAT (g)	1.3 ± 0.2	5.7 ± 0.4^&^	0.9 ± 0.1	6.8 ± 0.3^&^	0.8 ± 0.1	5.1 ± 0.5^&¤^
Visceral fat (% of BW)	4.6 ± 0.8	12.5 ± 0.5^&^	3.4 ± 0.5	14.1 ± 0.4^&^	3.4 ± 0.2	12.1 ± 0.7^&^
Body fat (% of BW)	6.1 ± 1.0	17.0 ± 0.5^&^	4.7 ± 0.4	17.8 ± 0.5^&^	4.6 ± 0.4	16.7 ± 1.0^&¤^

Values represent means ± s.e.m

*ND* normal diet, *HFD* high fat diet, *iWAT* inguinal fat (subcutaneous fat), *rWAT* peri-renal fat, *gWAT* peri-gonadal fat, *rWAT + gWAT* visceral fat. ^&^ *p* < 0.05 vs. corresponding genotype on *ND*; ^€^ *p* < 0.05 vs. Epac1^**−/−**^ *ND*; ^¤^ *p* < 0.05 vs. Epac1^**−/−**^ *HFD*

Body and fat weights were measured at the end of the feeding protocol, while the heart weight was measured after the ex vivo perfusions. Left ventricular (LV) volume was determined during the infarct size assessment

### Glucose dynamics

**Fig. 2:**
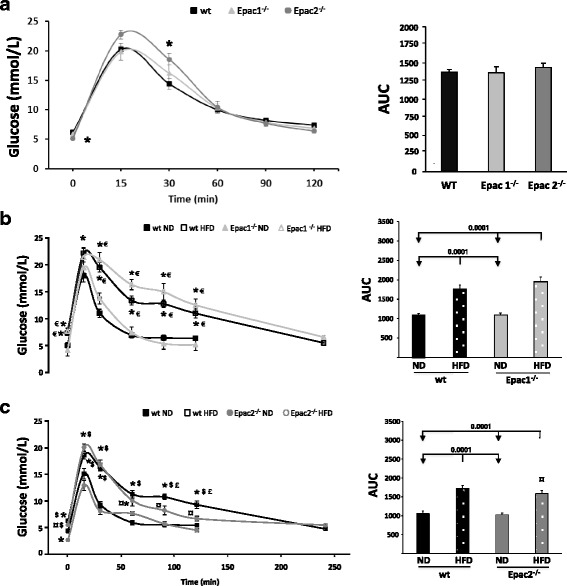
Glucose tolerance test after long-term feeding of an obesogenic high fat diet. Blood glucose levels during an intraperitoneal glucose tolerance test (ipGTT) in wild type (wt), Epac1 deficient (Epac1^−/−^) and Epac2 deficient (Epac2^−/−^) mice; before initiation of the feeding experiment (baseline) (**a**, **left panel**), and after 28 weeks of exposure to either a normal chow diet (ND) or a high fat diet (HFD) in Epac1^−/−^ (**b**, **left panel**) and Epac2^−/−^ mice (**c**, **left panel**). The area under the curves (AUC) indicate the total blood glucose variation during the ipGTT (**a-c, right panels**). Values are mean ± SEM. *p < 0.05 vs. wt *ND*. ^€^ *p* < 0.05 vs. Epac1^−/−^ *ND*. ^¤^ *p* < 0.05 vs. Epac1^−/−^ *HFD*. ^$^ *p* < 0.05 vs. Epac2^−/−^ *ND*. ^£^ *p* < 0.05 vs. Epac2^−/−^ *HFD*

An intraperitoneal glucose test (ipGTT) before HFD feeding revealed similar glucose dynamics for all cohorts (Fig. 2a, left panel). However, the Epac2^−/−^ group had slightly elevated blood glucose levels for the first 30min after injection, but their glucose level returned more rapidly to baseline, resulting in similar area under the curve (AUC) values for all groups (Fig. 2a, right panel).

Towards the end of the experimental feeding protocol (28 weeks), all genotypes exposed to long-term feeding of an obesogenic high fat diet had reduced ability to clear glucose and increased AUC as compared to the ND groups (Fig. 2b and c, left and right panels). The fasting blood glucose levels (0min) were significantly higher in the obese HFD groups as compared to the ND cohorts (wt *HFD* 7.4 ± 0.4 mmol/L vs. wt *ND* 5.2 ± 0.4 mmol/L, *p* < 0.05) (Epac1^−/−^ *HFD* 7.7 ± 0.5 mmol/L vs. Epac1^−/−^ *ND* 4.3 ± 0.2 mmol/L, *p* < 0.05) (Epac2^−/−^ *HFD* 6.5 ± 0.3 mmol/L vs. Epac2^−/−^ *ND* 3.2 ± 0.1 mmol/L, *p* < 0.05) (Fig. 2b and c, left panels). These data implicate that only the Epac2^−/−^ *HFD* group and the ND groups had euglycemic fasting blood glucose levels based on the human criteria for diabetes (≤7.0 mmol/L) (Fig. 2b and c, left panels). Furthermore, all groups fed a ND had glucose levels well within the euglycemic range (>7.8 mmol/L) at the end (120min) of the ipGTT. However, the Epac1^−/−^ *HFD* group (12.5 mmol/L) had glucose levels exceeding the human criteria for diabetes (>11.1 mmol/l), while the wt *HFD* (11.0 mmol/L) and Epac2^−/−^ *HFD* (7.8 mmol/L) can be considered as pre-diabetics with glucose levels between 7.8 and 11.0 mmol/L. After 240min, the blood glucose levels in all the HFD cohorts returned to normoglycemic levels (>7.8 mmol/L). The animals in the HFD cohorts had impaired glucose tolerance (longer time to clear a given amount of glucose), indicating deranged glucose homeostasis and reduced insulin sensitivity (Fig. 2b and c, right panels).

### Infarct size

**Fig. 3:**
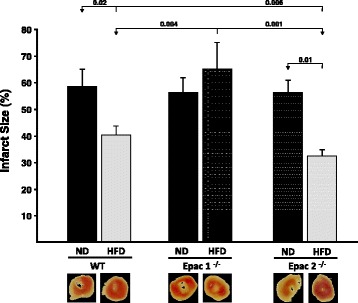
Myocardial tolerance to ischemia-reperfusion (I/R) injury after long-term feeding of an obesogenic high fat diet. Wild type (wt), Epac 1 (Epac1^−/−^) and Epac 2 (Epac2^−/−^) deficient mice were exposed to long-term feeding of either a normal chow diet (ND) or a high fat diet (HFD). At the end of the feeding protocol, the excised mice hearts were subjected to global ischemia (GI, 30min) and reperfusion (Rep, 60min) in a Langendorff perfusion model. Infarct size is expressed as percentage of the region at risk of infarction. Values are mean ± SEM and *n* ≥ 6. The numbers given in the figure indicate significance levels

In order to evaluate if long-term feeding of a HFD may exert cardioprotective properties, we subjected ex vivo mice hearts to 30min of global ischemia (GI) and 60min of reperfusion at the end of the feeding protocol (See Fig. 1a for feeding protocol and 1B for perfusion protocol). Infarct size, expressed as % of the ventricle, was significantly smaller in the wt obese (*HFD*) group compared to the wt normal diet (ND) group (wt HFD 41.3 ± 5.5% vs. wt ND 58.0 ± 9.8%, *p* < 0.05) (Fig. 3). Furthermore, to investigate if absence of Epac1 or Epac2 has an impact on IR-induced injury, we compared Epac1^−/−^ and Epac2^−/−^ mice to wt mice. Obese Epac2^−/−^ HFD mice had significantly smaller infarct size than the corresponding Epac2^−/−^ ND control group (Epac2^−/−^ *HFD* 34.4 ± 7.2% vs. Epac2^−/−^ *ND* 56.5 ± 3.8%, *p* < 0.05). In contrast, the Epac1^−/−^ mice had, if anything, larger infarcts when fed a HFD as compared a regular diet (Epac1^−/−^ *HFD* 65.1 ± 5.1% vs. Epac1^−/−^ *ND* 56.1 ± 3.5%, ns) (Fig. 3). Taken together, these results imply that Epac2 is not essential, but Epac1 may be required for the cardioprotection induced by long-term feeding of an obesogenic high fat diet.

### Cardiac functional recovery

**Fig. 4:**
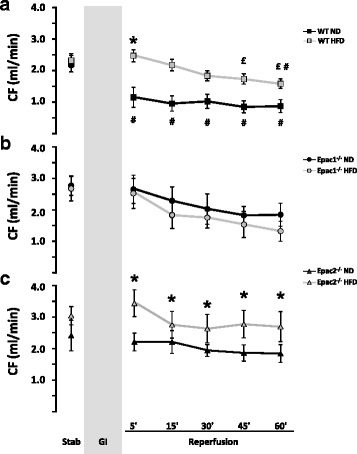
Coronary flow in the ex vivo perfused mice hearts. The coronary flow (CF) were registered after long-term feeding of a normal chow diet (ND) versus a high fat diet (HFD) in: **a** wild type (wt), **b** Epac1 deficient (Epac1^−/−^) and **c** Epac2 deficient (Epac2^−/−^) mice hearts. Values are mean ± SEM. ^*^ *p* < 0.05 vs. wt *ND*. ^£^ *p* < 0.05 vs. Epac2^−/−^ *HFD*. ^#^ *p* < 0.05 vs. corresponding pre-ischemic stabilization value

The post-ischemic coronary flow (CF) did not show consistent differences between the three genotypes, whether fed a normal or high fat diet (Fig. 4a-c), although wt *HFD* and Epac2^−/−^ *HFD* had borderline significantly elevated post-ischemic CF as compared to their corresponding ND groups (Fig. 4a and c).

**Fig. 5:**
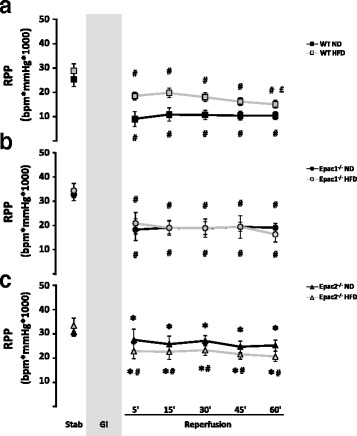
Cardiac rate-pressure product in the ex vivo perfused mice hearts. The rate-pressure product were calculated (RPP = LVSP x HR) at stabilization and during the post-ischemic reperfusion period in mice exposed to long-term feeding of a ND or HFD in: **a** wild type (wt), **b** Epac1 deficient (Epac1^−/−^) and **c** Epac2 deficient (Epac2^−/−^) mice hearts. Values are mean ± SEM. ^*^ *p* < 0.05 vs. wt *ND*. ^£^ *p* < 0.05 vs. Epac2^−/−^ *HFD*. ^#^ *p* < 0.05 vs. corresponding pre-ischemic stabilization value

All groups had significantly (*p* < 0.05) reduced post-ischemic rate-pressure product (RPP) compared to their corresponding stabilization value (Fig. 5a-c). There were no significant differences in RPP at reperfusion between the corresponding genotypes fed a ND versus a HFD (Fig. 5a-c), although RPP in the wt *HFD* tended to be higher than in the wt *ND* group (Fig. 5a).

**Fig. 6:**
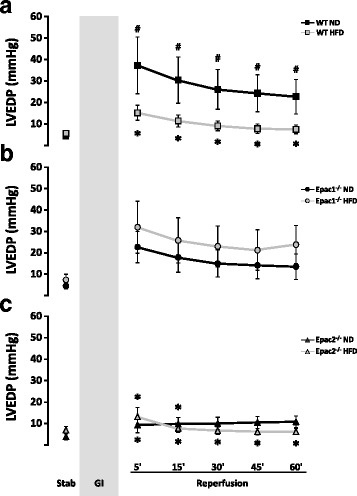
Cardiac left ventricular end-diastolic pressure in Langendorff perfused ex vivo mice hearts. Left ventricular end-diastolic pressure (LVEDP) were registered at stabilization and during the post-ischemic reperfusion period in hearts exposed to either a normal chow diet (ND) or a high fat diet (HFD) in: **a** wild type (wt), **b** Epac1 deficient (Epac1^−/−^) and **c** Epac2 deficient (Epac2^−/−^) mice hearts. Values are mean ± SEM. ^*^ *p* < 0.05 vs. wt *ND*. ^#^ *p* < 0.05 vs. corresponding pre-ischemic stabilization value

An elevated left ventricular end-diastolic pressure (LVEDP) indicate impaired contractility of the heart (contracture), presumably due to compromised calcium handling that may cause myocardial stunning, which subsides with prolonged reperfusion. LVEDP in the wt *ND* group was significantly (*p* < 0.05) elevated as compared to wt *HFD* and Epac2^−/−^ *HFD* during the 60min post-ischemic reperfusion period (Fig. 6a and c), in addition to being significantly higher than the corresponding pre-ischemic wt *ND* stabilization value (Fig. 6a). LVEDP in the Epac2^−/−^ *ND* group were significantly different from wt *ND* at 5 and 15min of reperfusion (Fig. 6a and c). There are no differences in LVEDP between the ND and HFD within the Epac1^−/−^ groups or within the Epac2^−/−^ groups during the reperfusion period (Fig. 6b and c).

All cohorts, except for the Epac2^−/−^ groups, presented with significantly (*p* < 0.05) reduced post-ischemic left ventricular developed pressure (LVDP) as compared to their corresponding stabilization values (Table 2). In regards to the maximum derivative of left ventricular pressure development (LV dP/dt_max_), the wt groups and the Epac^−/−^ *ND* showed a significantly lower post-ischemic recovery compared to their corresponding stabilization value (Table 2). We did not find any major differences between the groups in regards to HR and LV dP/dt_min_, except that the Epac1^−/−^ *HFD* group only had a significantly depressed LV dP/dt_min_ at 60min of reperfusion as compared to the corresponding stabilization value (Table 2).

**Table 2:** Hemodynamic parameters

	Group	Stab	5’ rep	15’ rep	30’ rep	45’ rep	60’ rep	Number
LVDP (mmHg)	wt *ND*	101 ± 7	56 ± 13^#^	63 ± 7^#^	56 ± 4^#^	54 ± 5^#^	51 ± 4^#^	8
	wt *HFD*	119 ± 8	94 ± 9^#^	104 ± 8	95 ± 5^*^	85 ± 5^#,*^	74 ± 4^#^	16
	Epac1^−/−^ *ND*	116 ± 4	72 ± 14^#^	73 ± 7^#^	75 ± 8^#^	77 ± 11^#^	71 ± 8^#^	6
	Epac1^−/−^ *HFD*	122 ± 14	89 ± 23^#^	81 ± 16^#^	76 ± 15^#^	82 ± 13^#^	69 ± 11^#^	6
	Epac2^−/−^ *ND*	101 ± 8	90 ± 8	92 ± 14	89 ± 12	87 ± 12	88 ± 13	6
	Epac2^−/−^ *HFD*	108 ± 7	81 ± 8	92 ± 12	91 ± 9	80 ± 8	78 ± 9	10
HR (bpm)	wt *ND*	255 ± 22	164 ± 28	173 ± 31	193 ± 29	204 ± 31	208 ± 27	8
	wt *HFD*	260 ± 20	215 ± 24	214 ± 24	196 ± 20^$^	195 ± 20	211 ± 24	16
	Epac1^−/−^ *ND*	268 ± 28	269 ± 44	271 ± 42	269 ± 45	272 ± 39	288 ± 39	6
	Epac1^−/−^ *HFD*	288 ± 24	259 ± 57	247 ± 38	254 ± 37	233 ± 43	244 ± 41	6
	Epac2^−/−^ *ND*	300 ± 39	313 ± 41	325 ± 60	338 ± 50	317 ± 51	315 ± 42	6
	Epac2^−/−^ *HFD*	316 ± 29	295 ± 33	280 ± 37	272 ± 27	288 ± 29	286 ± 27	10
dP/dt_min_ (mmHg/s)	wt *ND*	−3926 ± 629	−1287 ± 365^#^	−1549 ± 334^#^	−1511 ± 308^#^	−1642 ± 248^#^	−1954 ± 646^#^	8
	wt *HFD*	−4973 ± 363	−2742 ± 237^#^	−3096 ± 298^#^	−3024 ± 265^#*^	−2804 ± 239^#^	−2501 ± 183^#^	16
	Epac1^−/−^ *ND*	−4342 ± 325	−2378 ± 512^#^	−2408 ± 271^#^	−2464 ± 209^#^	−2458 ± 270^#^	−2457 ± 244^#^	6
	Epac1^−/−^ *HFD*	−4620 ± 691	−2633 ± 664	−2629 ± 553	−2522 ± 632	−2632 ± 539	−2205 ± 464^#^	6
	Epac2^−/−^ *ND*	−4325 ± 159	−3026 ± 342^#^	−2866 ± 298^#^	−3243 ± 260^#*^	−3106 ± 182^#^	−3284 ± 243^#^	6
	Epac2^−/−^ *HFD*	−5031 ± 715	−2997 ± 659^#^	−3352 ± 601^*^	−3074 ± 390^#*^	−3037 ± 381^#*^	−2771 ± 327^#^	10
dP/dt_max_ (mmHg/s)	wt *ND*	6327 ± 997	2701 ± 790^#^	3149 ± 648^#^	2717 ± 368^#^	2635 ± 297^#^	2465 ± 355^#^	8
	wt *HFD*	7195 ± 846	4632 ± 451^#^	5267 ± 549^#^	4784 ± 404^#^	4034 ± 353^#^	3731 ± 276^#^^$^	16
	Epac1^−/−^ *ND*	7208 ± 923	3486 ± 848^#^	3590 ± 490^#^	3875 ± 541^#^	3287 ± 445^#^	3414 ± 426^#^	6
	Epac1^−/−^ *HFD*	6522 ± 1226	4393 ± 1251	3916 ± 922	4038 ± 803	3844 ± 789	3377 ± 795	6
	Epac2^−/−^ *ND*	6958 ± 427	5586 ± 1082	5275 ± 865	5850 ± 310	4934 ± 363	6107 ± 275^*^	6
	Epac2^−/−^ *HFD*	6808 ± 935	4452 ± 617	5198 ± 771	4742 ± 691	4418 ± 629	4262 ± 661	10

^#^ *p* < 0.05 vs corresponding pre-ischemic stabilization value, ^*^ *p* < 0.05 vs. wt *ND*, ^$^ *p* < 0.05 vs. Epac2^−/−^ *ND*

## Discussion

Our findings can be summarised as follows: (1) Long-term feeding of a high-fat obesogenic diet (HFD) in wt mice increased their tolerance against ischemic-reperfusion (IR) induced injury, by significantly reducing infarct size in the ex vivo perfused mice heart. (2) The HFD did not afford cardioprotection in Epac1^−/−^ mice hearts, indicating involvement of Epac1-dependent signaling in HFD induced cardioprotection. (3) Epac2^−/−^ mouse hearts were, like those from wt animals, protected by long-term feeding of an obesogenic HFD. (4) The HFD intervention was not associated with compromised post-ischemic cardiac function or coronary flow.

### An obesogenic high fat diet enhances the tolerance against IR-induced injury

Our findings, summarized above, show that wt mice fed a HFD become more tolerant to acute myocardial ischemia. They are, thus, in line with recent human studies documenting reduced morbidity and mortality when investigating the impact of obesity on ischemic heart disease [9, 10, 30]. This “obesity paradox” is more frequently observed in the older patient population, coinciding with enhanced age-related risk of ischemic heart disease [31]. The paradox has also been described in type 2 diabetic patients with cardiovascular co-morbidity, in whom overweight and obesity may enhance survival in a setting of insulin-resistant diabetes [32]. Animal studies have confirmed the increased resistance to ischemic heart injury in obese, as well as lean, type 2 diabetic rats and rabbits [33–35], as well as in rats [11] and mice fed a HFD [36, 37]. Despite these findings, the existence and relevance of obesity-dependent cardioprotection remains controversial. Thus, other studies show increased myocardial susceptibility in mice with HFD-induced obesity [38, 39]. The conflicting results suggest that the effect of HFD-induced obesity may depend on subtle differences between the baseline activity and “setting” of signalling pathways at the time when ischemic stress is applied. There is little evidence that obesity acts on signaling pathways to promote cell survival. Obesity may, however, diminish the endothelial leakage with interstitial oedema formation, which is a major component of IR-injury (recently reviewed in [40]), through an increase of the signalling substance sphingosine 1 phosphate (S1P). S1P is increased in plasma of obese humans, genetically obese ob/ob mice and mice fed HFD [41]. Together with cAMP, S1P also mediates endothelial barrier tightening (reviewed in [42–45]). In addition, S1P has an acute cardioprotective effect in an in vivo murine IR-injury model [46] and has been found essential for successful ischemic postconditioning in the mouse heart [47].

### Epac1 mediates HFD induced cardioprotection

S1P and cAMP act together to maintain the endothelial barrier [42–45]. If endothelial junctional intactness is critical for the observed cardioprotection by HFD one will expect that deletion of Epac1, which maintains the endothelial barrier in vivo at basal S1P concentration [29], will diminish the protective effect of HFD. Since Epac2 does not affect the endothelial barrier [29], it is not expected to influence the protective effect of HFD through increased S1P. Although our results are in line with HFD protection through endothelial barrier tightening jointly with cAMP/Epac1, they do not rule out other possibilities, since Epac1 is expressed in cardiomyocytes, smooth muscle, and stromal fibroblasts [17, 18, 48, 49]. It is also likely that Epac1 is expressed in the epinephrine-secreting cells, as both adrenomedullary and chromaffin PC-12 cells express Epac1 [24].

Many previous studies have focussed on RISK (reperfusion injury salvage kinase) signaling as mediator of the protection against IR-induced injury [12, 13], and several groups have recently investigated the possible involvement of RISK dependent signaling in diet induced cardioprotection. Du Toit and coworkers showed that improved myocardial tolerance to IR- induced injury after 32 weeks of administration of an obesogenic diet was correlated with increased activation in baseline PKB/Akt, while they did not report on RISK activation at reperfusion [50]. However, a recent study by Lochner’s group did not confirm an association between infarct size reduction afforded by diet induced obesity and activation of the pro-survival RISK signaling components ERK p44/p42 and PKB/Akt [11]. In fact, the protected HFD group had the lowest activation of ERK and PKB/Akt at reperfusion. There is therefore no consistent evidence that HFD protection is primarily through modulation of the RISK pathway. Additional studies will have to further delineate the precise role of Epac1 in HFD induced protection. Mice with endothelial-specific deletion and ones with cardiomyocyte-specific deletion of Epac1 will be very useful to pinpoint the primary locus of the protective action of Epac1.

### Preserved contractile function and coronary flow with dietary high fat obesity

Long-term administration of a high-fat diet to wt mice improved post-ischemic coronary flow and functional recovery as compared to the wt ND fed group, corresponding to the reduced infarct size in the wt *HFD* group. The Epac2^−/−^ *HFD* group also presented with improved post-ischemic coronary flow and reduced infarct size. These data indicates that long-term feeding of an obesogenic high fat diet does not negatively influence myocardial contractile function in aged mice hearts, which are in agreement with experimental studies reported in the literature [11, 51]. However, there are reported discrepancies in terms of whether obesity exerts deleterious effects or preserves cardiac function. Obesity is an established independent risk factor for heart failure [52], though whether obesity directly promotes pump dysfunction is unknown. Some studies support impairment of systolic function [53, 54], whereas other data support preserved function in obese humans [55, 56]. Further studies are warranted to fully delineate long-term functional impact of a high fat diet and obesity on post-MI functional parameters.

There were no positive impact of feeding a high fat diet on functional recovery or coronary flow in Epac1-deficient mice hearts. Coronary flow is regarded as an important determinant of cardiac function, and it is possible that the decreased function reflect impaired flow due to hyperglycemia induced vascular or endothelial dysfunction, in addition to myocyte dysfunction. In our study, the overall glucose tolerance were comparable for all the HFD cohorts as indicated by similar AUCs. However, while there were no significant difference in the blood glucose levels between the HFD groups at the end of the ipGTT (120min), the Epac1^−/−^ *HFD* group presented with glucose levels slightly above the human diabetes criteria at this time point. Even so, the Epac1^−/−^ *HFD* group did not present with depressed cardiac function or coronary flow, neither at stabilization nor at post-ischemic reperfusion, as compared to the corresponding ND control group, thus indicating that the HFD did not have a detrimental effect on these functional endpoints. It is also noteworthy that the Epac1^−/−^ *HFD* hearts had increased heart weight compared to all other groups, possibly indicating a slight degree of hypertrophy. Increased muscle mass with reduced coronary flow could predispose for increased infarct size, but we do not consider this the main cause for the lack of cardioprotection in the Epac1^−/−^ *HFD* hearts, since both the heart-to-body weight ratio and heart volume did not differ from the other HFD cohorts.

## Conclusion

The results of the present study demonstrate that wild type mice made obese by long-term feeding of a high fat diet have enhanced tolerance towards ischemic-reperfusion (IR) induced injury. Furthermore, the HFD failed to afford cardioprotection in Epac1^−/−^ mice hearts, indicating that HFD mediated cardioprotection require Epac1-dependent signaling, which therefore may implicate a mediating role for Epac1 in the so called “obesity paradox”.

## Abbreviations

8-CPT: 8-pCPT-2’-O-Me-cAMP
AMI: Acute myocardial infarction
AUC: Area under the curve
BW: Body weight
cAMP: cyclic adenosine monophosphate
CF: Coronary flow
Epac proteins: Exchange Proteins directly Activated by cAMP; Epac1 and Epac2; also called cAMPGEFs
Epac1 (Epac1^−/−^): Epac1 knock out mouse
Epac2 (Epac2^−/−^): Epac2 knock out mouse
GDP: Guanosine diphosphate
GI: Global ischemia
GTP: Guanosine triphosphate
HFD: High fat diet
HR: Heart rate
HW: Heart weight
IPC: Ischemic preconditioning
ipGTT: intraperitoneal glucose tolerance test
IR: Ischemia reperfusion
KHB: Krebs-Henseleit buffer
LVDP: Left ventricular developed pressure
LVdP/dt_max_: Maximum derivative of left ventricular pressure development
LVdP/dt_min_: Minimum derivative of left ventricular pressure development
LVEDP: Left ventricular end-diastolic pressure
ND: Normal chow diet
RISK: Reperfusion injury salvage kinase pathway
RPP: Rate pressure product
Wt: Wild type
β-PC: β-adrenergic preconditioning
